# Supplementary material for: Dendritic cell MST1 inhibits Th17 differentiation
Source: Nat Commun. 2017 Feb 1;8:14275. doi: 10.1038/ncomms14275 (PMC5296641; doi:10.1038/ncomms14275)
Supplement: Supplementary Information — Supplementary Figures and Supplementary Table [file ncomms14275-s1.pdf]

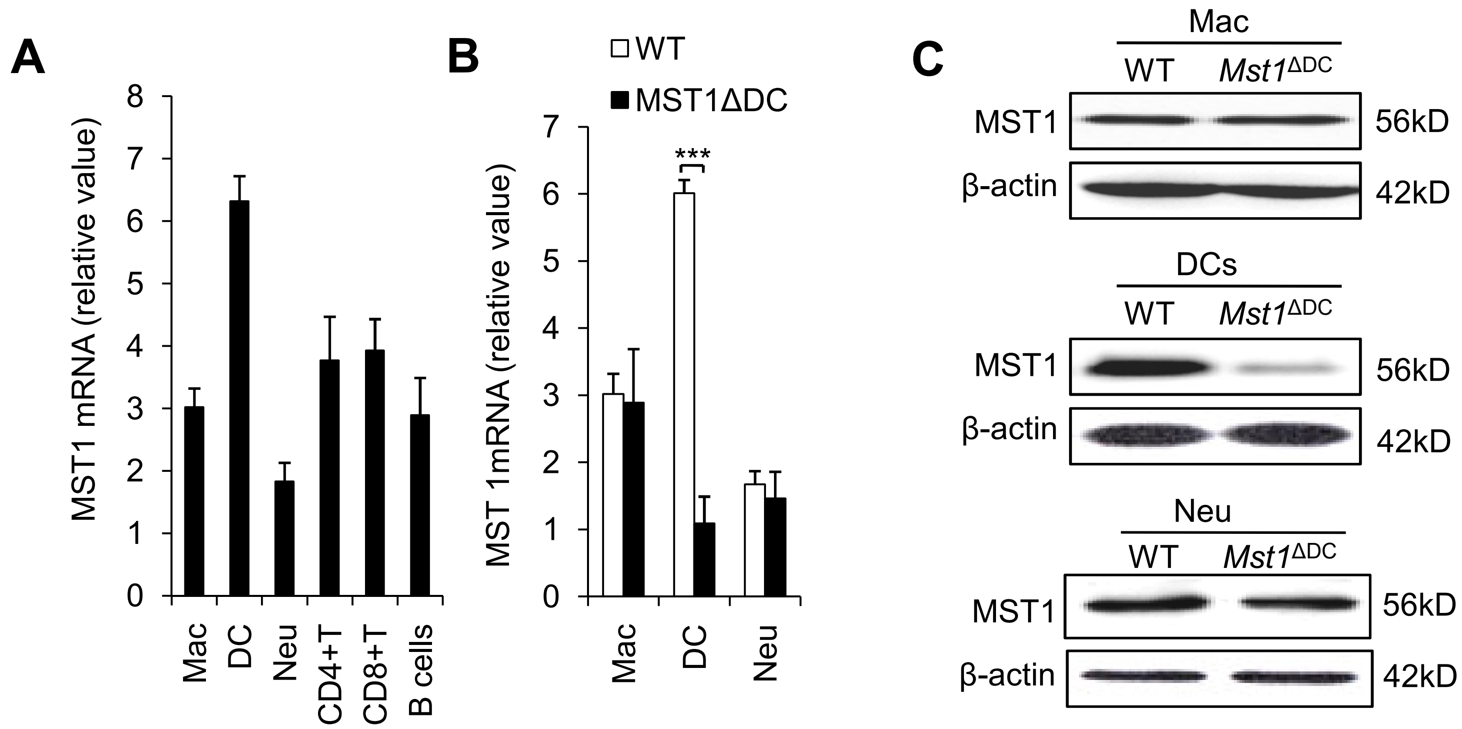

Supplementary Figure 1. **MST1 expressions in different immune cell populations in WT or *Mst1*$^{\Delta DC}$ mice**.

Macrophages (CD11b^{+}F4/80^{+}), DCs (CD11c^{+}MHCII^{+}F4/80^{-}Ly6G^{-}NK1.1^{-}CD19^{-}TCR^{-}), neutrophils (CD11b^{+}Ly6G^{+}), CD4^{+}T cells (CD4^{+}TCR^{+}), CD8^{+}T cells (CD8^{+}TCR^{+}) and B cells (CD19^{+}) were isolated from mouse spleen and the purity was confirmed by flow cytometry. Indicated cells were isolated from spleen in wild-type (WT) or *Mst1*$^{\Delta DC}$ mice and MST1 expressions were determined by qPCR analysis (Levels based on the ratio to HPRT1; **A-B**). **C**, Indicated cells were isolated and the MST1 expression were determined by western blot. Data are shown as mean ± s.d., n=5, from one of two independent experiments. ***P<0.001, compared with the indicated groups. P values were determined using Student's t-tests.

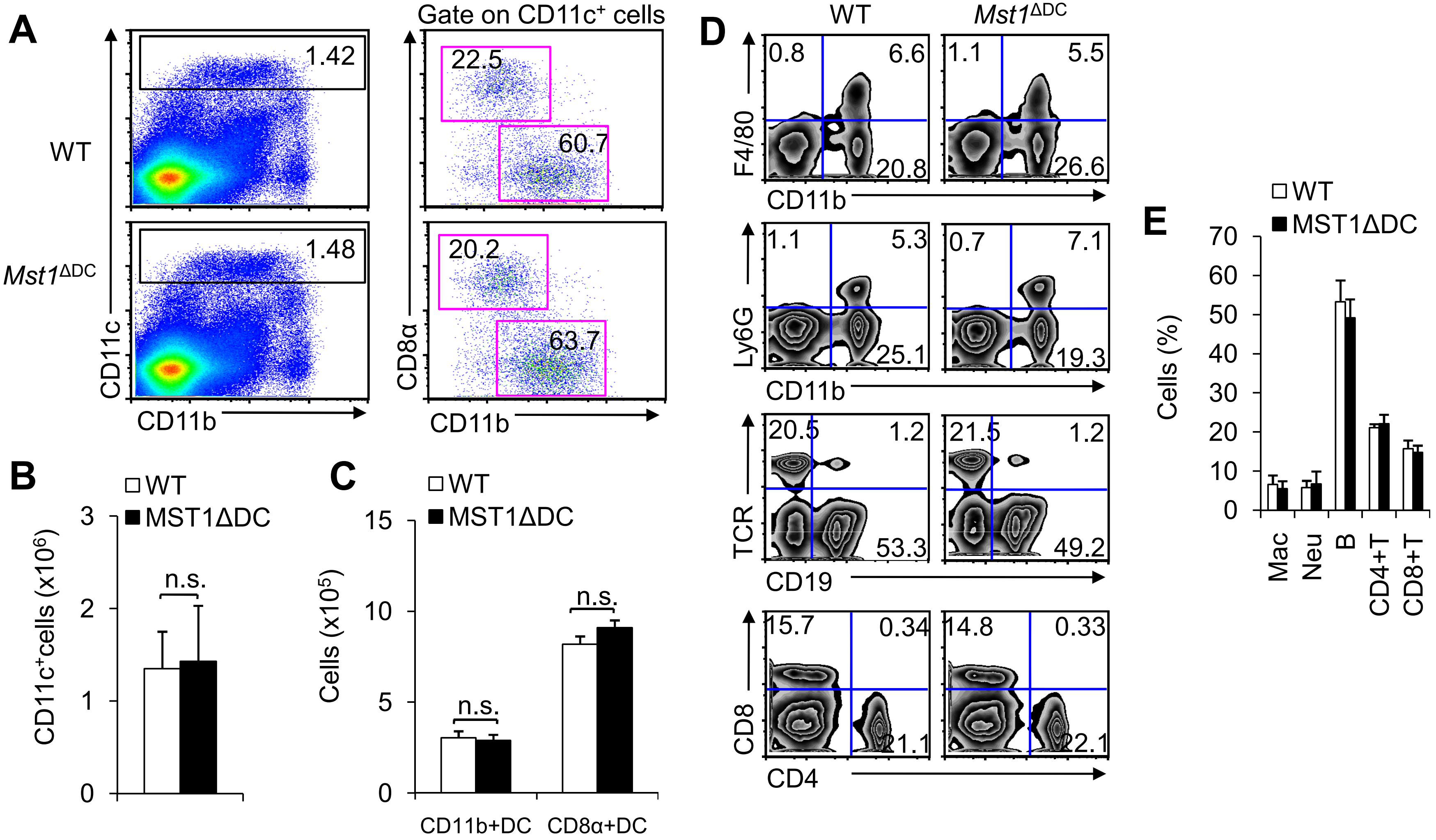

Supplementary Figure 2. MST1 deficiency in DC does not affect the development and homeostasis of immune cells.
(**A**) Flow cytometry analysis of $CD11b^+CD11c^+$DCs. (**B-C**) The absolute cell number of $CD11c^+$ cells (B) and $CD11b^+$DC or $CD8\alpha^+$DC (C) in spleen from WT and *Mst1*$^{\Delta DC}$ mouse is summarized. (**D-E**) Flow cytometry analysis of $CD11b^+F4/80^+$ macrophages (Mac), $CD11b^+Ly6G^+$ neutrophils (Neu), $CD19^+$ cells (B cells), $CD4^+TCR^+$ cells (CD4⁺T) and $CD8^+TCR^+$ cells (CD8⁺T)) in spleen from WT and *Mst1*$^{\Delta DC}$ mouse and typical figure is shown in **D**, and data summarized in **E**. Data are shown as mean $\pm$ s.d., n=4-5, from one of two independent experiments. n.s., not significant. *P* values were determined using Student's *t*-tests.

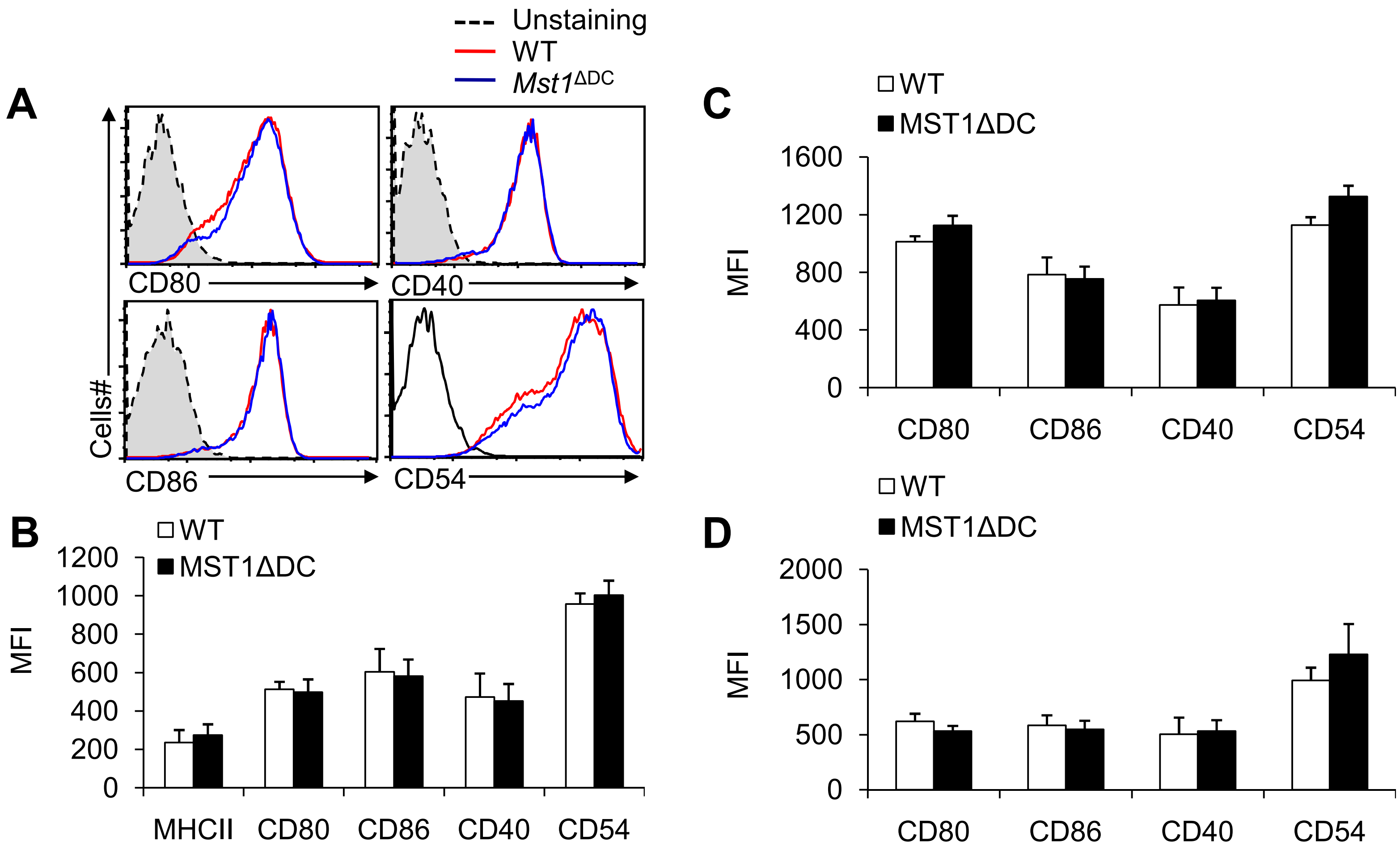

Supplementary Figure 3. MST1 deficiency in DC does not alter the expressions of co-stimulatory molecules of DCs.
(**A**) Flow cytometry analysis of expressions of the co-stimulatory molecules in $CD11b^{+}CD11c^{+}$DCs. (**B**) The mean fluorescent intensity (MFI) of co-stimulatory molecules in DCs is summarized. (**C**) MFI of co-stimulatory molecules in DCs stimulated by LPS (**C**; 100 ng/ml) for 12 h *in vitro* is summarized. (**D**) Sorted Naïve $CD4^{+}$T cells from OT-II mice stimulated with antigen and DCs from WT or *Mst1*$^{\Delta DC}$ mice for 3 d. The MFI of co-stimulatory molecules in DCs is summarized. Data are shown as mean ± s.d., n=4-5, from one of three independent experiments.

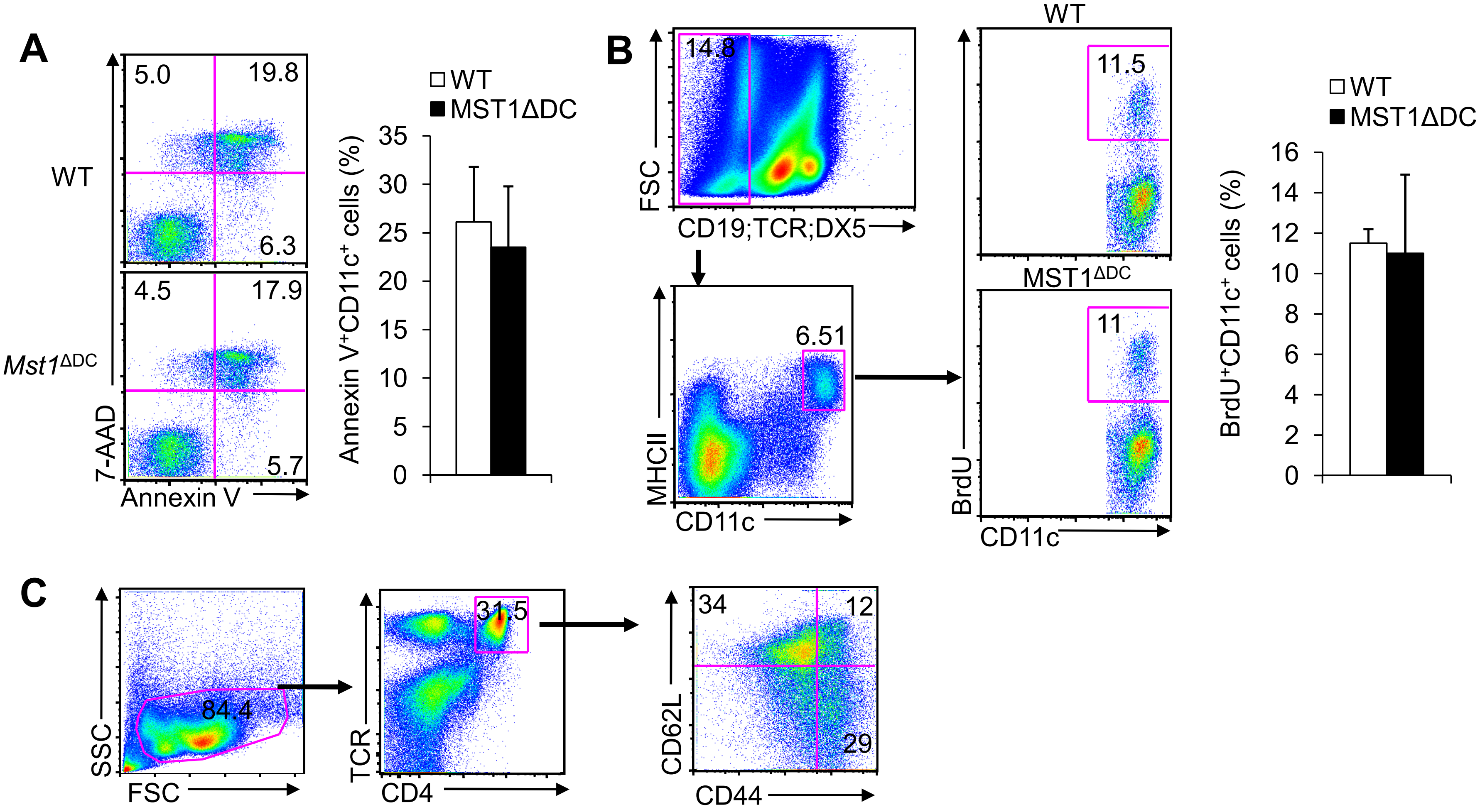

Supplementary Figure 4. MST1 deficiency in DC does not alter the apoptosis and proliferation of DCs.
(**A**) The frequency of apoptotic and dead CD11c⁺ cells was assessed with Annexin V and 7-AAD staining. Typical figure is shown in left and data summarized in the right. (**B**) For the *in vivo* BrdU incorporation experiments, 150 µl of a 10-mg/ml BrdU solution was injected intraperitoneally into mice for 96 h. Spleen was isolated from indicated mice, and BrdU⁺ cells were visualized with the BrdU flow kit from BD Biosciences PharMingen using an FITC-labeled anti-BrdU antibody. Samples were also stained with CD11c-PE to visualize DCs. Typical figure shown in left and data summarized in right. Data are shown as mean ± s.d., n=4-5, from one of three independent experiments. (**C**) Gating strategies for determining the percentage of $CD4^{+}TCR^{+}CD44^{high}CD62L^{low}$ cells.

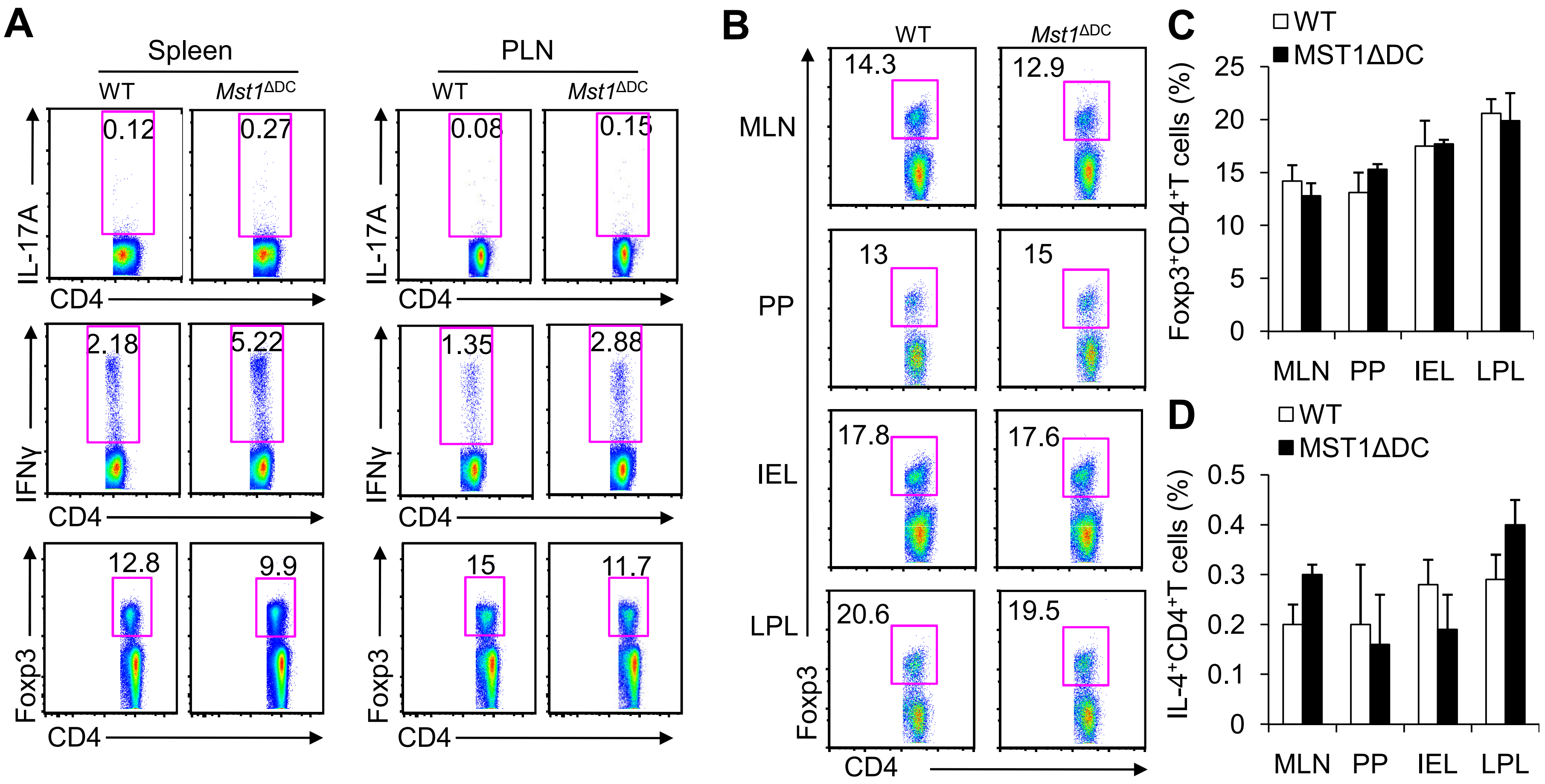

Supplementary Figure 5. Expressions of cytokines and transcriptional factors of CD4⁺T cells in *Mst1*ΔDC and WT old-aged mice.

Intracellular IL-17A, IFNγ and Foxp3 expressions of CD4⁺T cells in spleen and PLN of WT and *Mst1*ΔDC mice at 20 weeks after birth and typical figure shown (**A**). Foxp3 and IL-4 expression of CD4⁺T cells isolated from MLN, PP, IEL and LPL in WT and *Mst1*ΔDC mice at 20 weeks after birth and typical figure is shown (**B**) and the frequencies of positive cells are summarized (**C-D**). Data are shown as mean ± s.d., n=4-8, from one of three to four independent experiments.

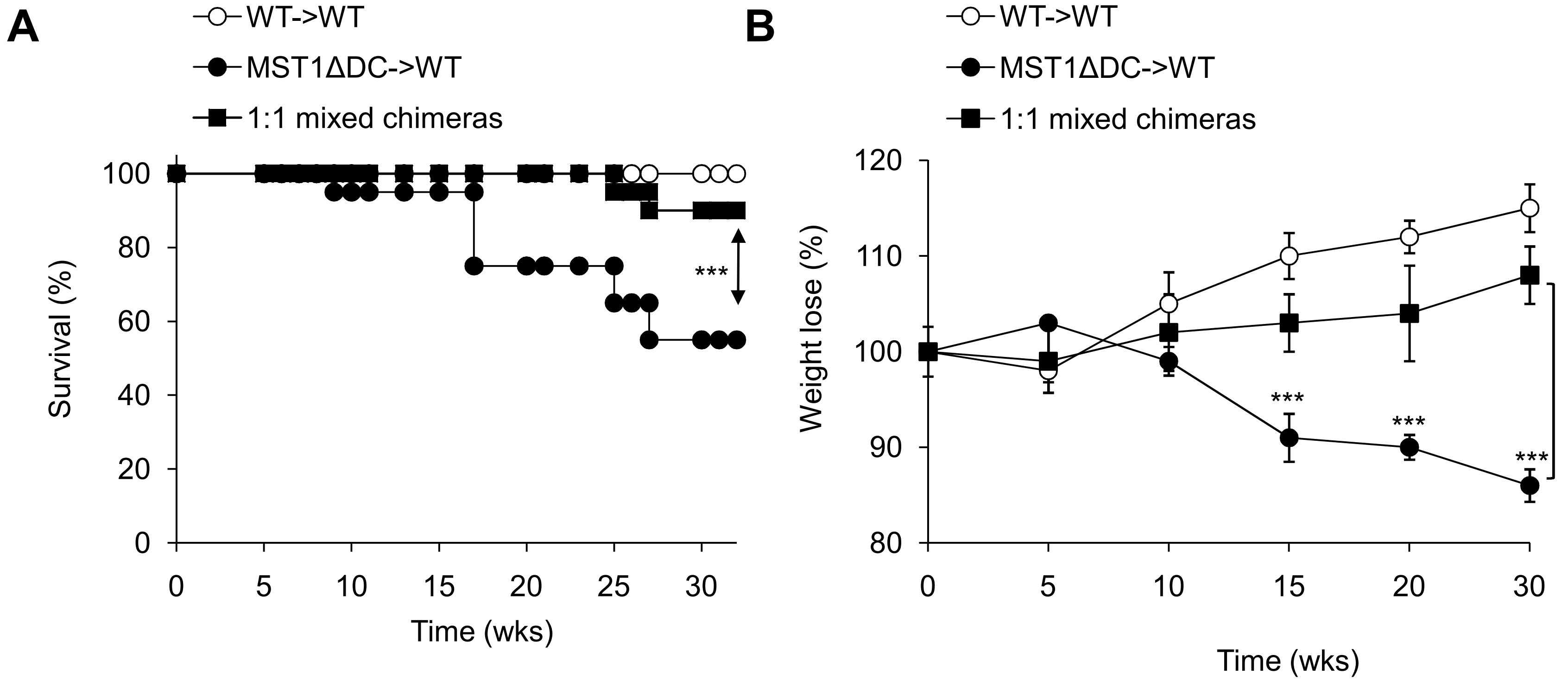

Supplementary Figure 6. Survival and weight changes in *Mst1*$^{\Delta DC}$ and WT chimeras mice.
Kaplan-Meier plots of mouse survival (**A**) and body weight (**B**) from WT→WT chimera mice, *Mst1*$^{\Delta DC}$→WT chimera mice and 1:1 *Mst1*$^{\Delta DC}$–WT mixed chimera mice is summarized (n=20).

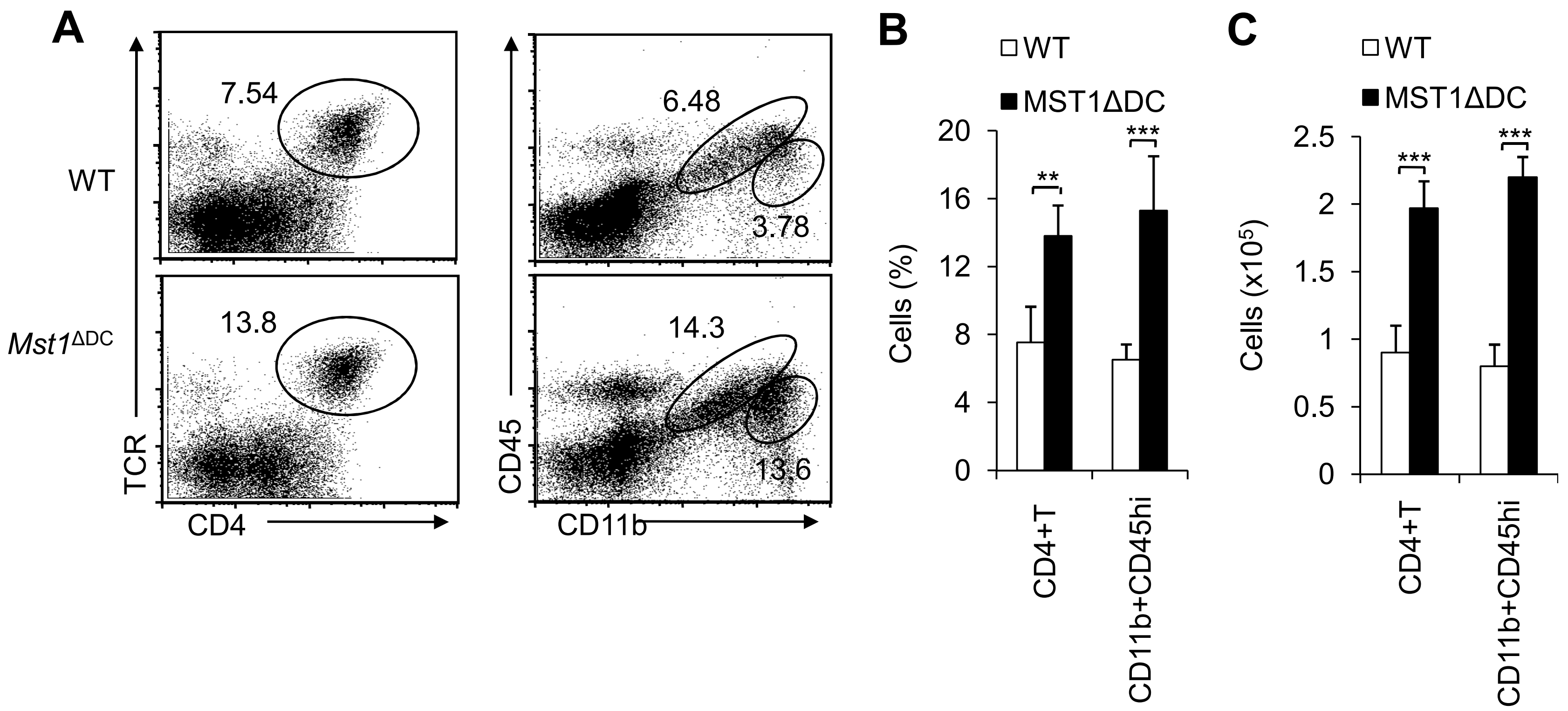

Supplementary Figure 7. DC MST1 deficiency promotes inflammatory cell infiltration in EAE.
(**A**) The surface staining of $CD4^{+}TCR^{+}$ cells, $CD11b^{+}CD45^{high}$ cells from spinal cords of WT or *Mst1*$^{\Delta DC}$ mice were determined with FCM on day 19 after MOG immunization. The percentage of positive cells (**B**) and absolute cell number (**C**) are summarized. Data are representative of three independent experiments (mean ± s.d.; n=4-6). ***P*<0.01; ****P*<0.001, compared with the indicated groups. *P* values were determined using Student's *t*-tests.

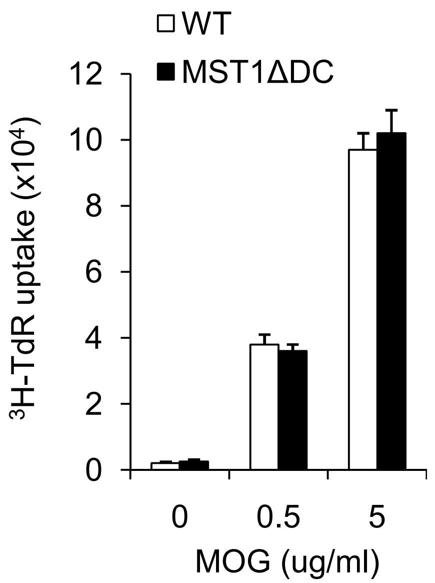

Supplementary Figure 8. DC MST1 deficiency does not alter the T cell proliferation in EAE.
CD4$^+$T cells isolated from draining lymph nodes (dLN) of MOG-immunized WT or *Mst1*$^{\Delta DC}$ mice were stimulated with MOG for 5 days. T cell proliferation was determined with ^{3}H-TdR incoporation as described in Method. Data are representative of three independent experiments (mean ± s.d.; n=3-4).

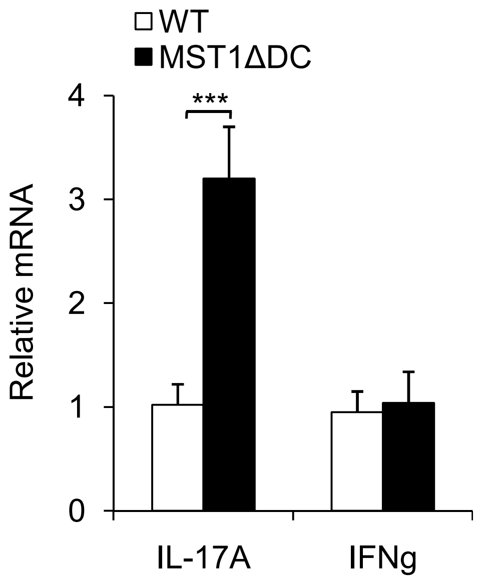

Supplementary Figure 9. DC MST1 signaling directs Th17 differentiation *in vivo*.
MOG-trangenic 2D2 T cells (Thy1.1$^+$) were transferred into WT or *Mst1*$^{\Delta DC}$ mice and immunized with MOG+CFA for 7-8 days. (**A**) The mRNA expression of IL-17 and IFNγ in donor cells stimulated with MOG for 72 h. Level in WT groups were set to1. Data are representative of three independent experiments (mean ± s.d.; n=4-6). ***P<0.001, compared with the indicated groups. P values were determined using Student's t-tests.

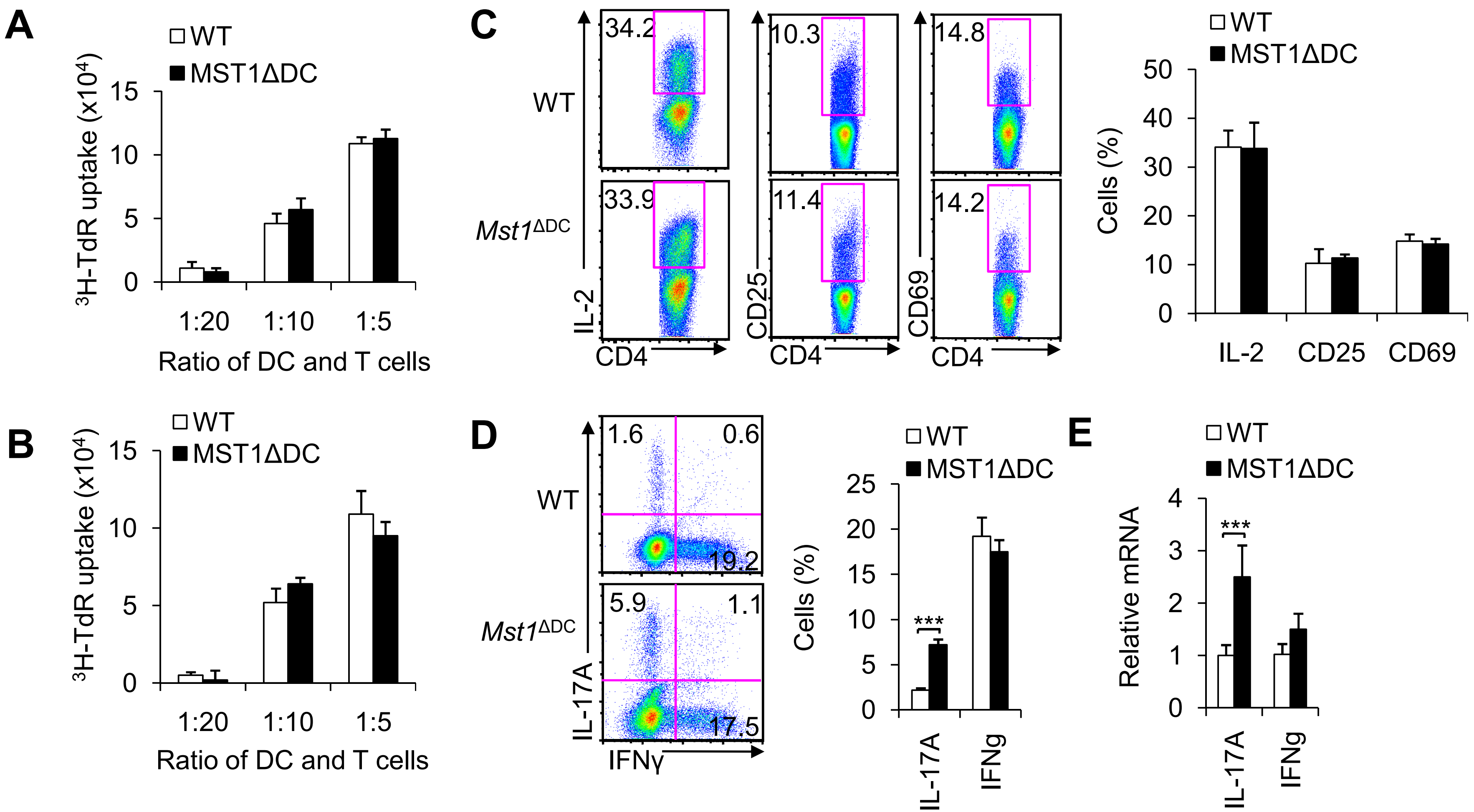

Supplementary Figure 10. DC MST1 signaling programming Th17 differentiation *in vitro*.

(**A**) Sorted Naïve CD4$^+$T cells from MOG-trangenic 2D2 T cells stimulated with antigen (MOG, 5 ug/ml) and splenic DCs from WT or *Mst1*$^{\Delta DC}$ mice at the indicated ratio for 5 d. T cell proliferation was determined with ^{3}H-TdR incoporation as described in Method. (**B-C**) Sorted Naïve CD4$^+$T cells from OT-II mice stimulated with antigen (OVA, 5 ug/ml) and splenic DCs from WT or *Mst1*$^{\Delta DC}$ mice at the indicated ratio for 5 d. T cell proliferation was determined with ^{3}H-TdR incoporation as described in Method (**B**) and expressions of IL-2, CD25 and CD69 in CD4$^+$T cells were analyzed by FCMs, typical figure shown (**C**; left) and data is summarized (**C**; right). (**D-E**) Sorted Naïve CD4$^+$T cells from OT-II mice stimulated with antigen and LPS-pulsed splenic DCs from WT or *Mst1*$^{\Delta DC}$ mice for 5 d. (**D**)The intercellular staining of IL-17 and IFNγ in T cells. Right, the proportion of IL-17 and IFNγ in T cells. (**E**) The mRNA expressions of indicated genes in T cells were determined with qPCR. Data are representative of three independent experiments (mean ± s.d.; n=3-4). ***P<0.001, compared with the indicated groups. P values were determined using Student's t-tests.

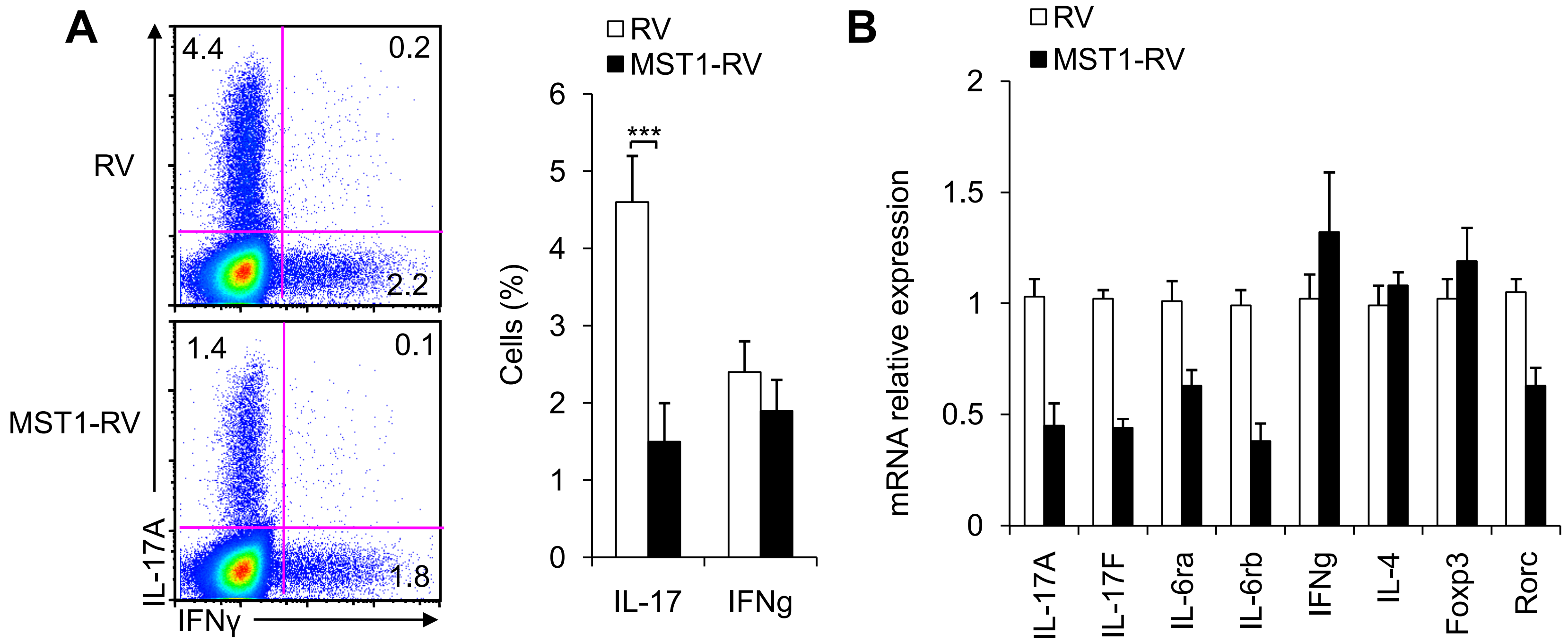

Supplementary Figure 11. DC MST1 signaling programming Th17 differentiation *in vitro*.
Ectopically expressed MST1 with a retrovirus and sorted the positive green fluorescent protein (GFP) DCs and co-cultured with OT-II cells in the presence of antigen and LPS for 5 d. (**A**)The intercellular staining of IL-17 and IFNγ in T cells. Right, the proportion of IL-17 and IFNγ in T cells. (**B**) The mRNA expressions of indicated genes in T cells were determined with qPCR. Data are representative of three independent experiments (mean ± s.d.; n=3-4). ***P<0.001, compared with the indicated groups. P values were determined using Student's t-tests.

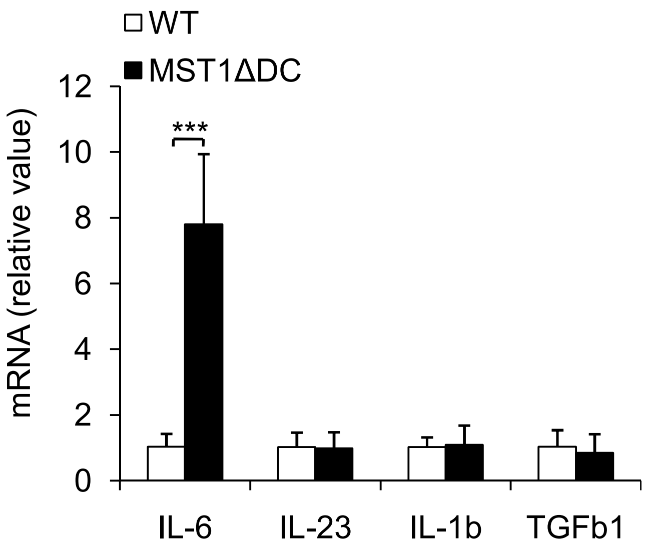

Supplementary Figure 12. DC MST1 signaling controls IL-6 production *in vitro*.
mRNA expression of indicated genes in WT or *Mst1*$^{\Delta DC}$ splenic DCs following LPS stimulation *in vitro*. Levels in WT control groups were set to 1. Data are representative of three independent experiments (mean ± s.d.; n=3). ****P*<0.001, compared with the indicated groups. *P* values were determined using Student's *t*-tests.

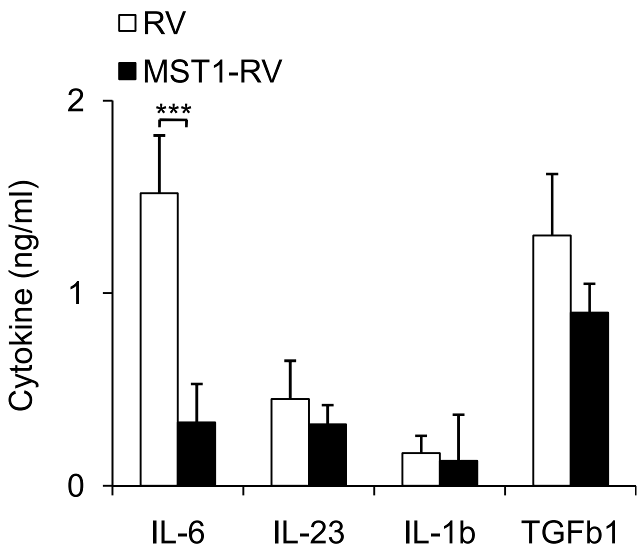

Supplementary Figure 13. DC MST1 signaling programming Th17 differentiation *in vitro*.
Ectopically expressed MST1 with a retrovirus and sorted the positive green fluorescent protein (GFP) DCs were stimulated with LPS for 24 h. The supernatant was collected and indicated cytokine secretion were determined with ELISA. Data are representative of three independent experiments (mean ± s.d.; n=3-5). ***$P<0.001$, compared with the indicated groups. *P* values were determined using Student's *t*-tests.

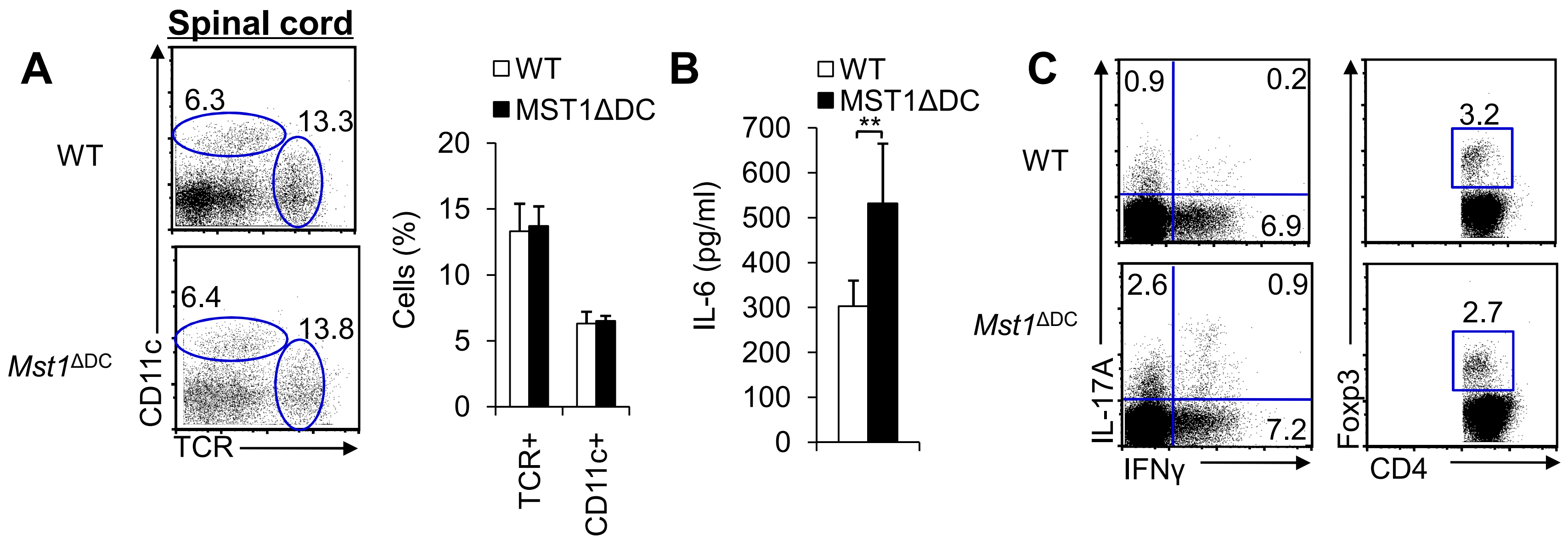

Supplementary Figure 14. Spinal cord DC MST1 signaling programming Th17 differentiation *in vitro*.

(**A**) The surface staining of TCR$^+$ cells and CD11c$^+$ cells from spinal cords of WT or *Mst1*$^{\Delta DC}$ mice were determined with FCM. Typical figure shown in the left and the percentage of positive cells is summarized in the right. WT or *Mst1*$^{\Delta DC}$ DCs isolated from spinal cords 5 h following LPS stimulation *in vitro*. Supernatant was collected and cytokine secretions were determined with ELISA (**B**). (**C**) Sorted Naïve CD4$^+$T cells from C57BL/6 mice stimulated with anti-CD3 (2 ug/ml) and LPS-pulsed spinal cord DCs from WT or *Mst1*$^{\Delta DC}$ mice for 5 d. Intercellular staining of IL-17, IFNγ and Foxp3 in T cells were determined with FCM and typical figure shown. Data are representative of three independent experiments (mean ± s.d.; n=4). ***P*<0.01, compared with the indicated groups. *P* values were determined using Student's *t*-tests.

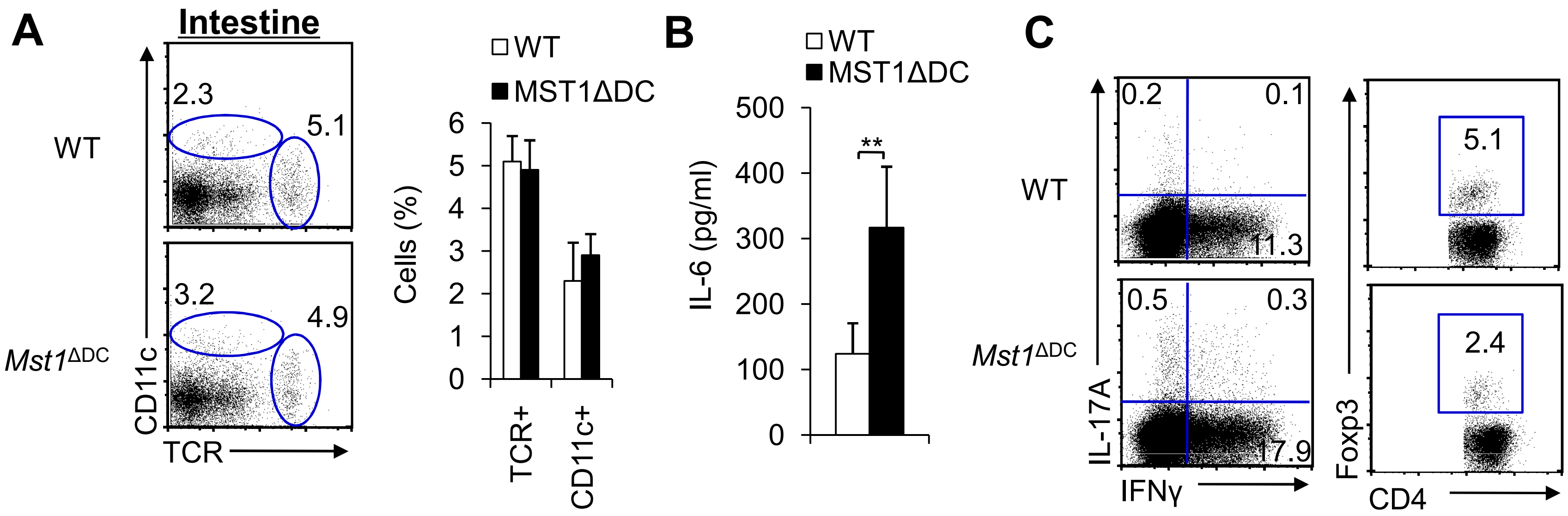

Supplementary Figure 15. Intestinal DC MST1 signaling programming Th17 differentiation *in vitro*.
(**A**) The surface staining of TCR⁺ cells and CD11c⁺ cells from intestine of WT or *Mst1*$^{\Delta DC}$ mice were determined with FCM. The typical figure shown in the left and the percentage of positive cells is summarized in the right. WT or *Mst1*$^{\Delta DC}$ DCs isolated from intestine 5 h following LPS stimulation *in vitro*. Supernatant was collected and cytokine secretions were determined with ELISA (**B**). (**C**) Sorted Naïve CD4^{+}T cells from C57BL/6 mice stimulated with anti-CD3 (2 ug/ml) and LPS-pulsed intestinal DCs from WT or *Mst1*$^{\Delta DC}$ mice for 5 d. Intercellular staining of IL-17, IFNγ and Foxp3 in T cells were determined with FCM and typical figure shown. Data are representative of three independent experiments (mean ± s.d.; n=5-6). ***P*<0.01, compared with the indicated groups. *P* values were determined using Student's *t*-tests.

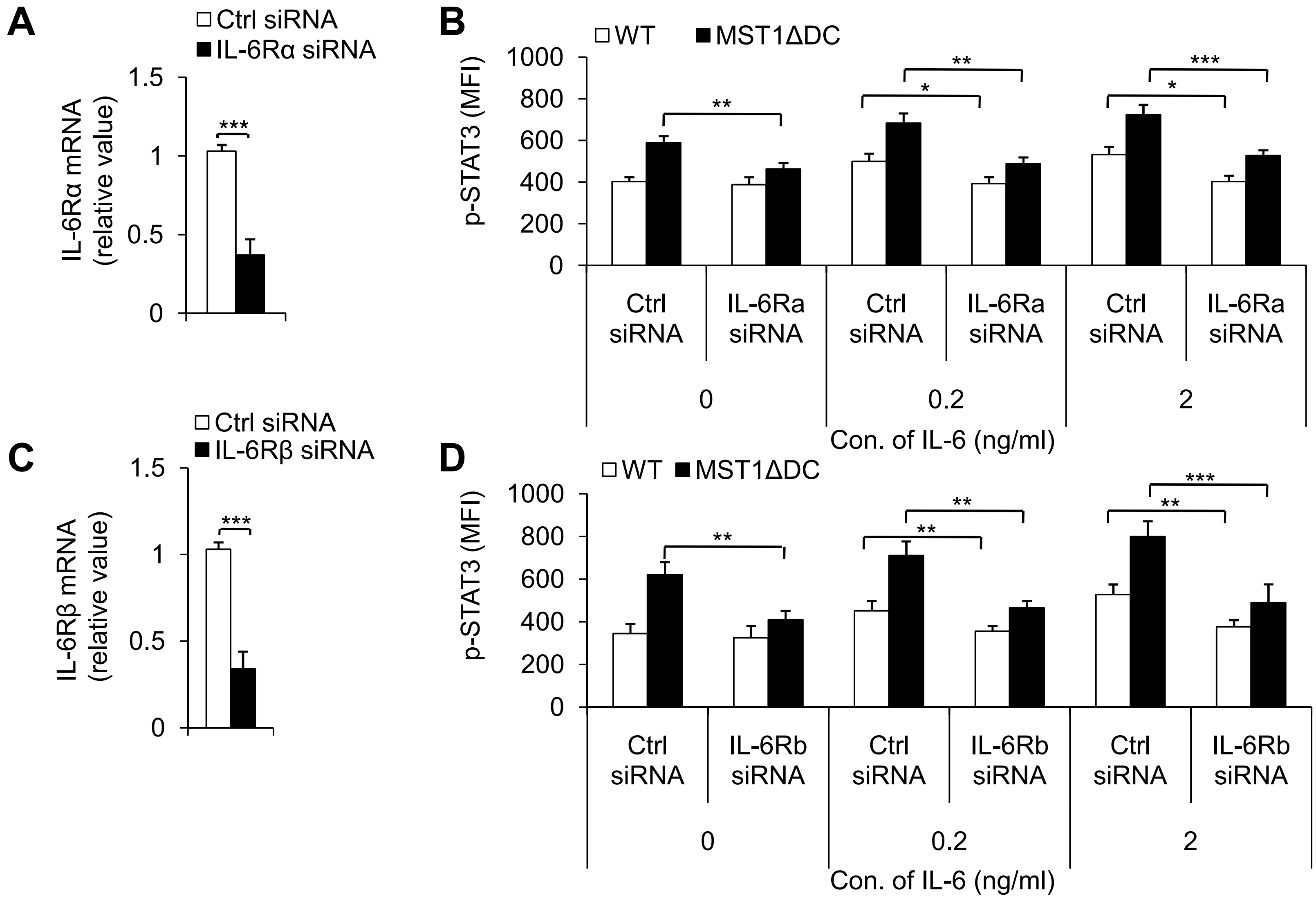

Supplementary Figure 16. IL-6R-dependent phosphorylation of STAT3 of T cells is responsible for MST1 signaling in DC. Sorted CD4⁺ T cells were transfected with control or IL-6Rα siRNA vector (**A**-**B**) or IL-6Rβ siRNA vector (**C**-**D**) and stimulated with WT or *Mst1*^{ΔDC} splenic DCs for 5 days in the presence of different concentration of IL-6. Expression of indicated mRNA (**A** or **C**) were determined with qPCR (Levels of ctrl siRNA were set to 1). (**B** or **D**) The intercellular staining of p-STAT3 in T cells were determined with FCM and the MFI of p-STAT3 in T cells. Data are representative of three independent experiments (mean ± s.d.; n=3). *P<0.05, **P<0.01, and ***P<0.001compared with the indicated groups. P values were determined using Student's t-tests.

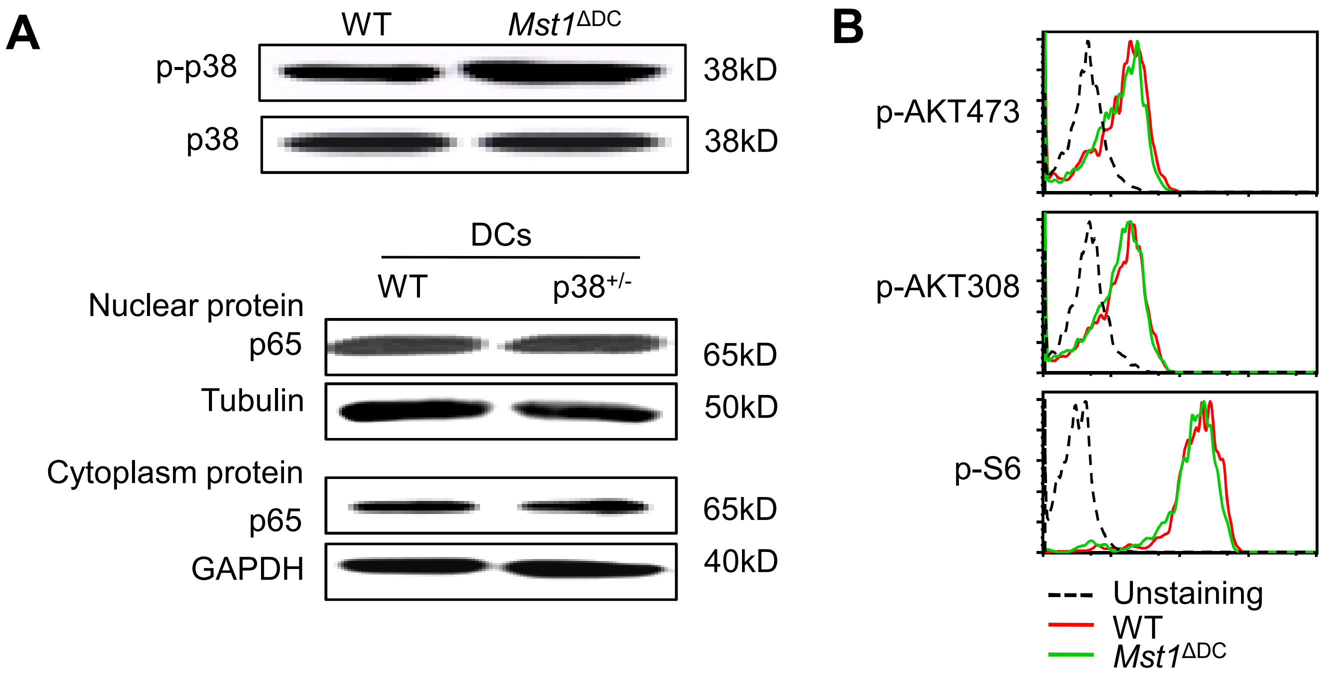

Supplementary Figure 17. Expressions of p38MAPK, NF-kB and AKT-mTOR signals in DC cells.
(**A**) Immunoblot of the indicated proteins in splenic DCs following LPS (100 ng/ml) stimulation for 60 min *in vitro*. (**B**) Flow cytometry of the indicated proteins in splenic DCs following LPS (100 ng/ml) stimulation for 60 min *in vitro*.

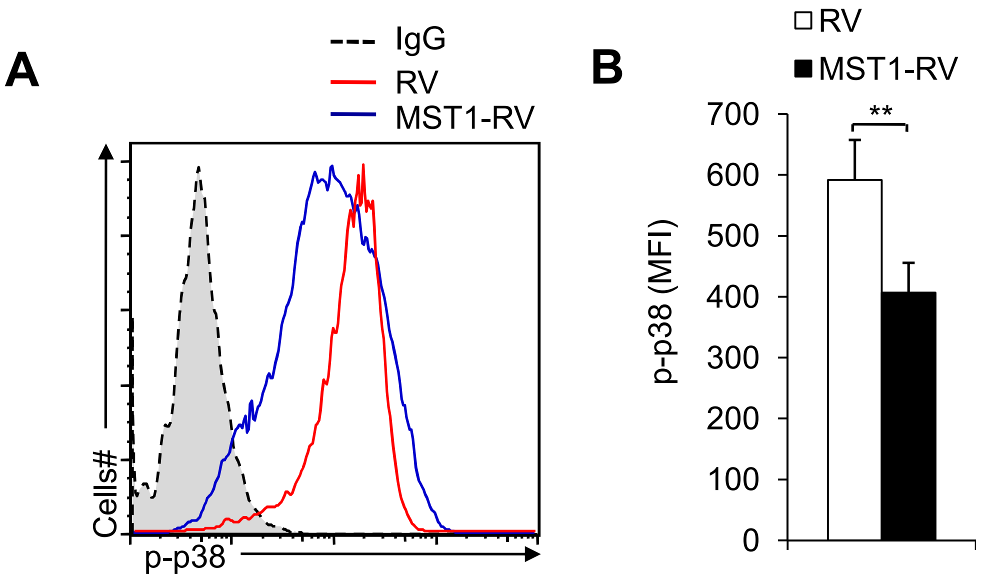

Supplementary Figure 18. Modest expression of MST1 in DC downregulates the activation of p38MAPK.
Ectopically expressed MST1 with a retrovirus and sorted the positive green fluorescent protein (GFP) DCs were stimulated with LPS for 120 mins. (**A**)The intercellular staining of p-p38MAPK. (**B**) MFI of p-p38 in T cells. Data are representative of three independent experiments (mean ± s.d.; n=3-4). ***P*<0.01 compared with the indicated groups. *P* values were determined using Student's *t*-tests.

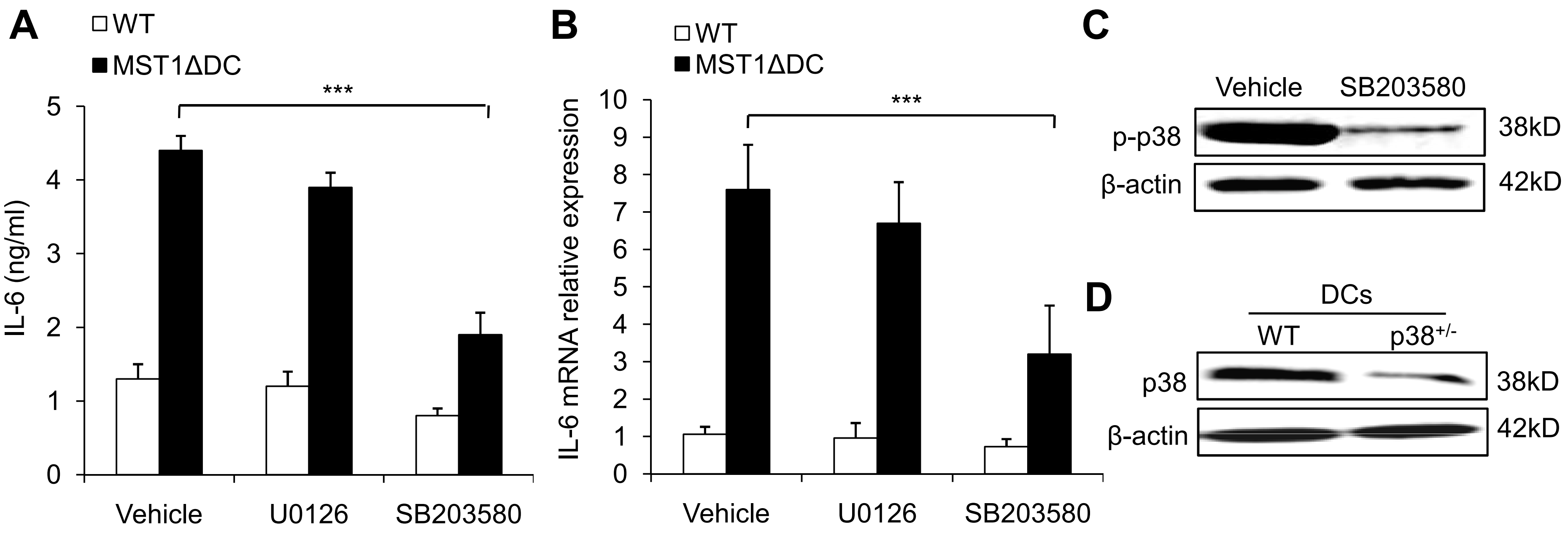

Supplementary Figure 19. MST1 regulates IL-6 production through p38MAPK.
WT and *Mst1*$^{\Delta DC}$ DCs were pretreated with U0126 (10 uM) and SB203580 (5 uM) 30-60 mins and stimulated by LPS (10 ng/ml) for 5 h. IL-6 secretion (**A)** and IL-6 mRNA expression (**B**) were determined with ELISA and qPCR. The mRNA levels of WT groups were set to 1. (**C**) Immunoblot of p-p38 in DCs with the indicated treatment. (**D**) Immunoblot of p38 in DCs. Data are representative of three independent experiments (mean ± s.d.; n=3-4). ****P*<0.001 compared with the indicated groups. *P* values were determined using Student's *t*-tests.

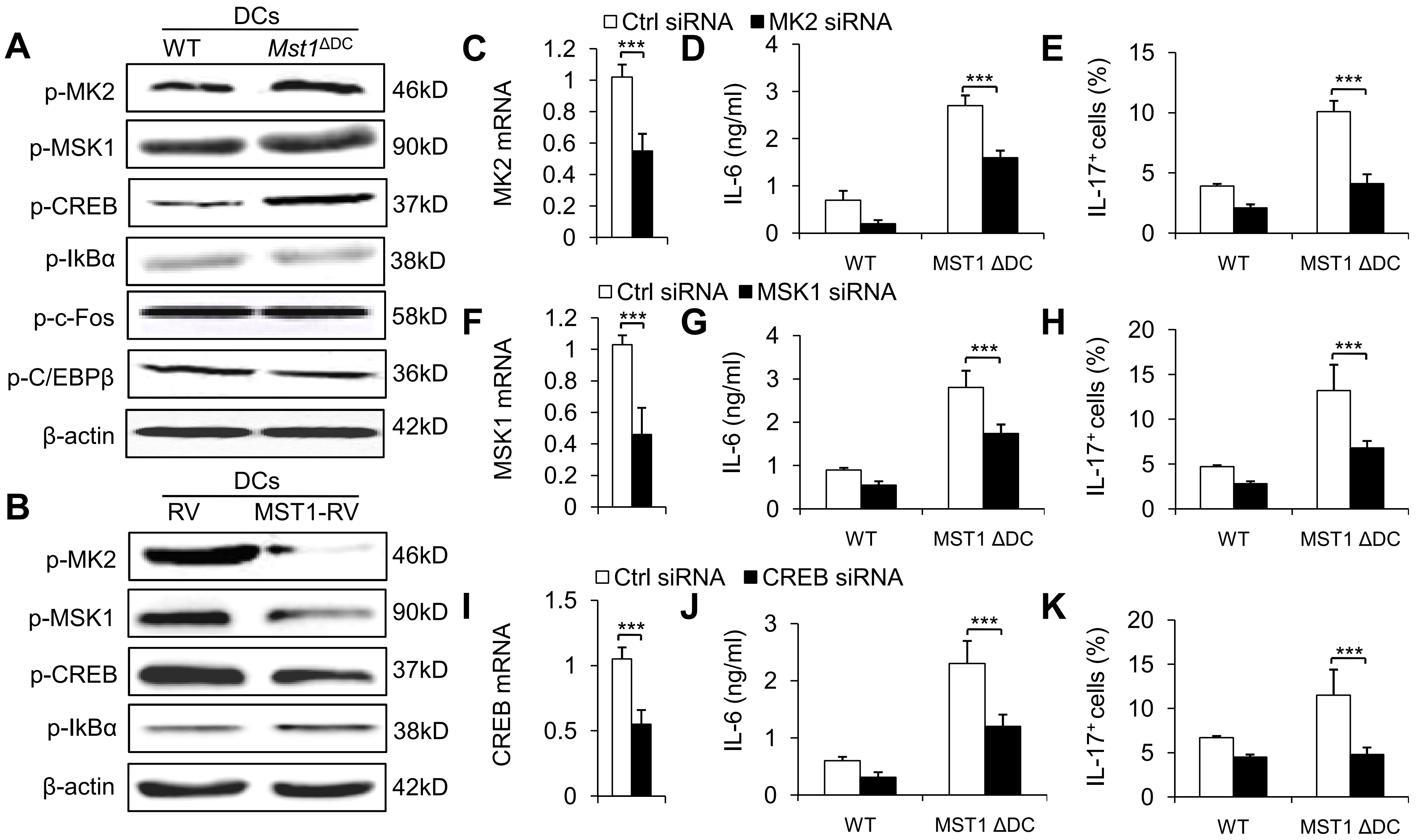

Supplementary Figure 20. Roles of MK2, MSK1 and CREB on IL-6 production in MST1 deficient DC cells.
(**A**) Immunoblot of indicated proteins in splenic DCs from *Mst1*$^{\Delta DC}$ mice or WT mice following LPS (100 ng/ml) stimulation for 30 min. (**B**) Immunoblot of indicated proteins in GFP^{+}DC transduced with RV or MST1-RV and stimulated by LPS for 30 min. Expression of indicated mRNA (**C**, **F** or **I**) and IL-6 production (**D, G** or **J**) by DCs transfected with control siRNA (Ctrl siRNA) or indicated siRNA and stimulated for 5 h following LPS (100 ng/ml) (Expressions in Ctrl siRNA were set to 1). Sorted CD4^{+} T cells stimulated with WT or *Mst1*$^{\Delta DC}$ splenic DCs for 5 d. Intercellular staining of IL-17 in T cells were determined with FCM and data is summarized in (**E**, **H**, or **K**). Data are representative of three independent experiments (mean ± s.d.; n=3-5). ****P*<0.001 compared with the indicated groups.

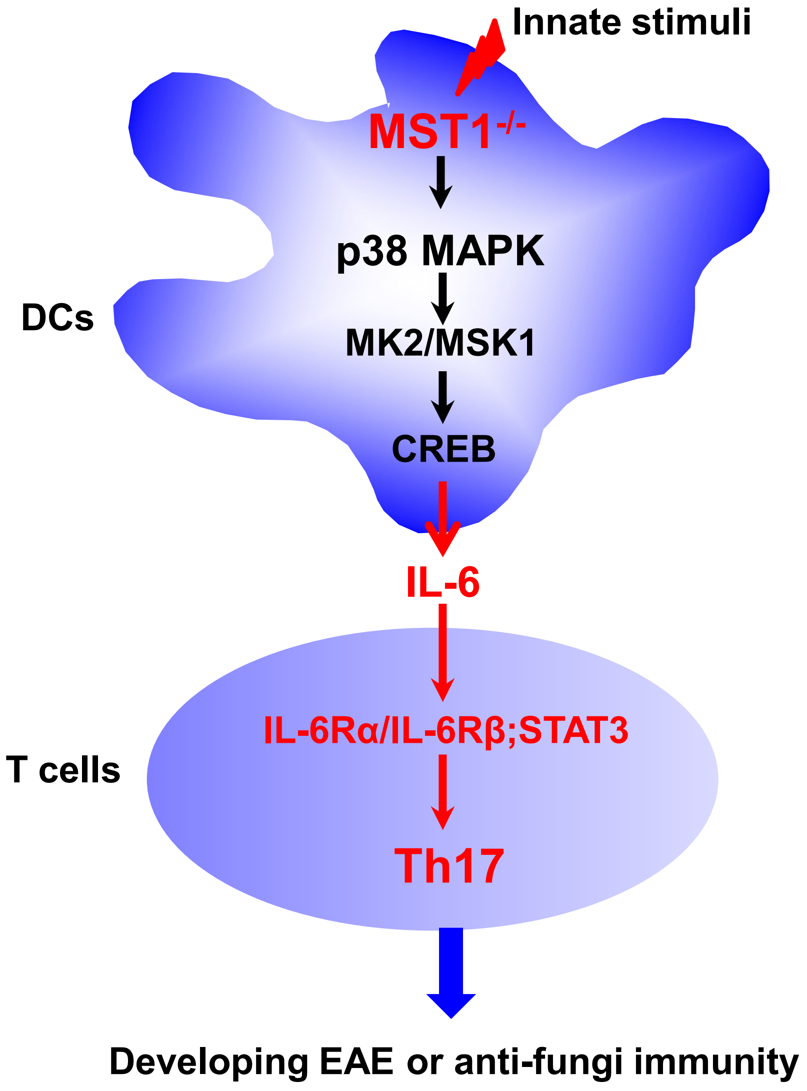

Supplementary Figure 21. DC MST1 controls Th17 differentiation in autoimmune and fungal infectious inflammation. Proposed model of how MST1 in DCs integrates innate stimuli to regulate the differentiation of Th17 populations in diseases.

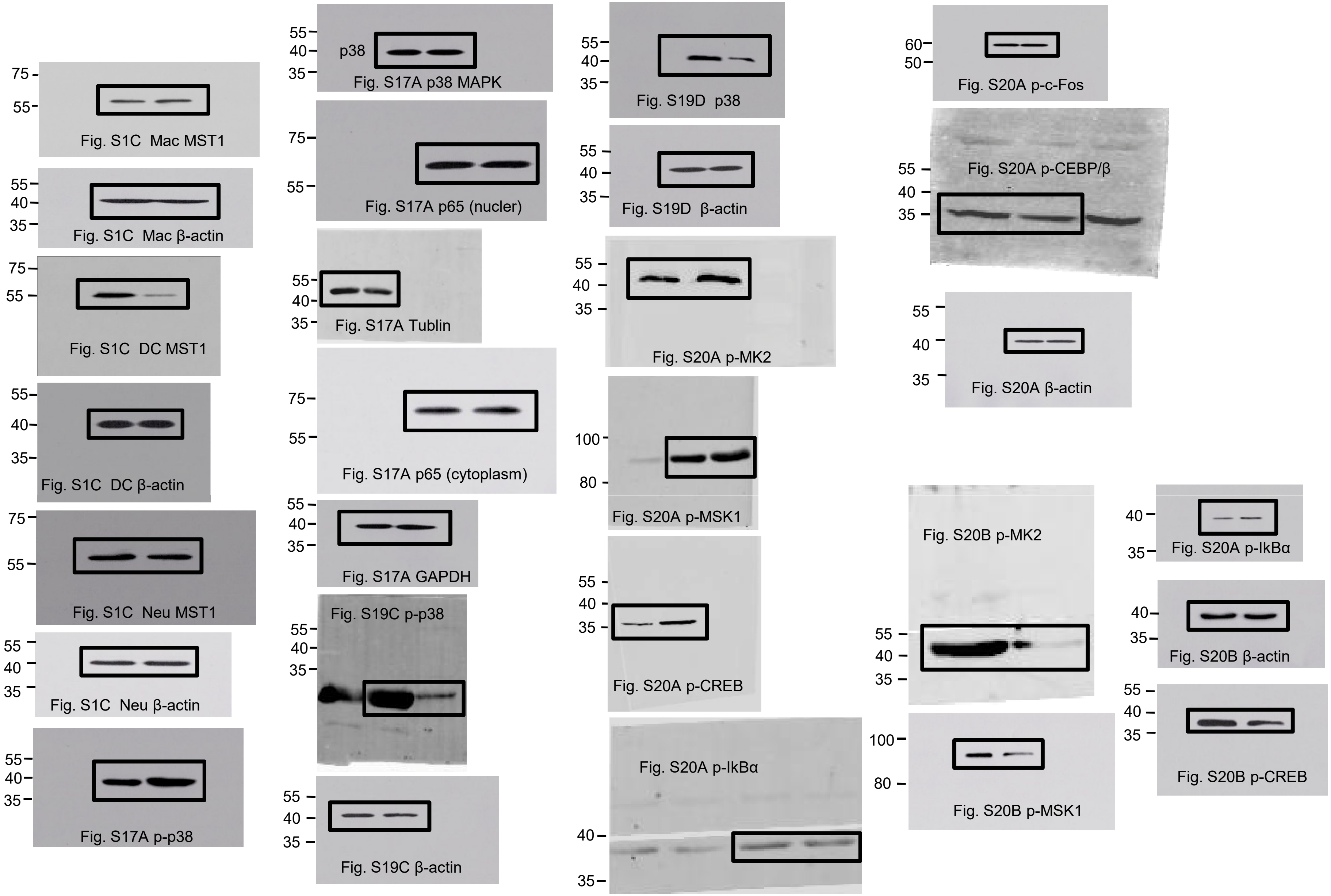

Supplementary Figure 22. Original western blots for images used in Fig. S1, Fig. S17, Fig. S19 and Fig. S20. Each antibody produced a clear band at the expected size. Blocks indicate the specific bands used in the figure.

Supplementary Table 1. Primer sequences used for qPCR assays.

Genes	Forward primer	Reverse primer
Foxp3	5'-GGCCCTTCTCCAGGACAGA-3'	5'-GGCATGGGCATCCACAGT-3'
Mst1	5'-CGAATTCTCACATCGTGCCAGAATCCATTTC-3	5′-GCGAATTCTTAAGTTCCTTCCTCTTCCTCATCCTC-3′
hMst1	5′-TTTTAGTTTGTGAAATGGGATTTAGGATTT-3′	5′-CCATACCCRTCCTCTTAACCAATAA-3′
Rora	5'-GACCCACACCTCACAAATTGA-3'	5'-AGTAGGCCACATTACACTGCT-3'
Rorc	5'-CCGCTGAGAGGGCTTCAC-3'	5'-TGCAGGAGTAGGCCACATTACA-3'
T-bet	5'-AGCAAGGACGGCGAATGTT-3'	5'-GGGTGGACATATAAGCGGTTC-3'
IFNγ	5'-TCGAATCGCACCTGATCACTA-3'	5'-GGGTTGTTCACCTCGAACTTG-3'
IL-4	5'-CCACGGATGCGACAAAAAT-3'	5'-TCTCCTGTGACCTCGTTCAAAA-3'
IL-6Rα	5′-CATTGCCATTGTTCTGAGGTTC-3′	5′-AGTAGTCTGTATTGCTGATGTC-3′
hIL-6Rα	5'-TGC TGA CCA GTC TGC CAG GAG A-3'	5'-ACA CTA CTG GCG ACG CAC ATG-3'
IL-6Rβ	5'-TCCCATGGGCAGGAATATAG-3'	5'-CCATTGGCTTCAGAAAGAGG-3'
hIL-6Rβ	5'-TGT TGA CGT TGC AGA CTT GG-3'	5'-TCT GGA GGC AAG CCT GAA AT-3'
IL-17A	5′-CTC CAG AAG GCC CTC AGA CTA C-3′	3′-GGG TCT TCA TTG CGG TGG-5′
IL-17F	5′-GAG GAT AAC ACT GTG AGA GTT GAC-3′	5′-GAG TTC ATG GTG CTG TCT TCC-3′
mHPRT	5'-CCTAAGATGAGCGCAAGTTGAA-3'	5'-CCACAGGACTAGAACACCTGCTAA-3'
hHPRT	5'-CAGTATAATCCAAAGATGGTCAA-3'	5'-TTAGGCTTTGTATTTTGCTTTTCC-3'
GATA3	5'-GAATCCTCTGCATCAACAAGC-3'	5'-GGGCAAGGGTTCTGAGGT-3'
GAPDH	5'-GACTTCAACAGCAACTCCCAC-3'	5'-TCCACCACCCTGTTGCTGTA-3'
